# Aerobic Exercise and Pharmacological Therapies for Skeletal Myopathy in Heart Failure: Similarities and Differences

**DOI:** 10.1155/2016/4374671

**Published:** 2016-01-19

**Authors:** Aline V. Bacurau, Telma F. Cunha, Rodrigo W. Souza, Vanessa A. Voltarelli, Daniele Gabriel-Costa, Patricia C. Brum

**Affiliations:** School of Physical Education and Sport, University of São Paulo, 05508-030 São Paulo, SP, Brazil

## Abstract

Skeletal myopathy has been identified as a major comorbidity of heart failure (HF) affecting up to 20% of ambulatory patients leading to shortness of breath, early fatigue, and exercise intolerance. Neurohumoral blockade, through the inhibition of renin angiotensin aldosterone system (RAS) and *β*-adrenergic receptor blockade (*β*-blockers), is a mandatory pharmacological therapy of HF since it reduces symptoms, mortality, and sudden death. However, the effect of these drugs on skeletal myopathy needs to be clarified, since exercise intolerance remains in HF patients optimized with *β*-blockers and inhibitors of RAS. Aerobic exercise training (AET) is efficient in counteracting skeletal myopathy and in improving functional capacity and quality of life. Indeed, AET has beneficial effects on failing heart itself despite being of less magnitude compared with neurohumoral blockade. In this way, AET should be implemented in the care standards, together with pharmacological therapies. Since both neurohumoral inhibition and AET have a direct and/or indirect impact on skeletal muscle, this review aims to provide an overview of the isolated effects of these therapeutic approaches in counteracting skeletal myopathy in HF. The similarities and dissimilarities of neurohumoral inhibition and AET therapies are also discussed to identify potential advantageous effects of these combined therapies for treating HF.

## 1. Introduction

Heart failure (HF) is a significant cause of morbidity and mortality associated with high health care costs [1]. More than 20 million people worldwide are estimated to have HF, and this situation is more critical considering that the prevalence of HF will rise as the mean age of the population increases [1].

Cardiac cachexia is a serious complication of HF with a prevalence of 16–42% [2] and associated with loss of appetite (anorexia), anemia, systemic inflammation, altered hormones, metabolic abnormalities, and skeletal myopathy. Altogether, these features lead to severe and unintentional body weight loss that occurs in cachectic states. Skeletal myopathy is one of the main features of cardiac cachexia associated with HF progression and severity. It is characterized by abnormalities in skeletal muscle structure and function that include atrophy, a shift toward fast twitch fibers, muscle metabolic dysfunction, and impaired muscle contractility that, combined, play a major role in shortness of breath, early fatigue, and exercise intolerance observed in HF [3–6].

Chronic muscle underperfusion and/or metabolic disturbance in HF lead to an overactivation of muscle afferents, such as mechano-metaboreceptors [7, 8], exacerbating sympathetic nervous system. Importantly, sustained sympathetic hyperactivity adversely affects muscle performance in HF by altering its metabolic status and limiting oxygen supply to exercising muscle contributing to exercise intolerance [9]. Furthermore, sustained sympathetic hyperactivity increases muscle reactive oxygen species (ROS) production (e.g., by catecholamine autooxidation and activation of nicotinamide adenine dinucleotide phosphate-oxidase-NADPH oxidase) and muscle inflammation [9–15], which contributes to skeletal myopathy in HF [10]. Altogether, these alterations will set up a vicious cycle between skeletal myopathy and progression of HF [6] being addressed as “muscle hypothesis” [16–18]. In fact, the clinical course of HF involves a continuous compensatory activation of neurohumoral systems, such as renin angiotensin aldosterone (RAS) system and sympathetic nervous system paralleled by parasympathetic withdrawal [19].

The understanding of HF as a neurohumoral disorder instead of a hemodynamic disease (throughout most of the 20th century) has driven changes in the mandatory pharmacological therapy of HF. In this sense, inhibition of the RAS and *β*-adrenergic receptor antagonists (*β*-blockers) has gained strength in HF therapy reducing symptoms, mortality, and sudden death. However, the impact of these drugs on skeletal myopathy needs to be clarified, since HF patients optimized with *β*-blockers and inhibitors of RAS still display, to certain degree, skeletal myopathy and exercise intolerance.

It is widely recognized that aerobic exercise training (AET) is an efficient nonpharmacologic therapy for HF that improves quality of life and exercise tolerance; the latter associated with remarkable attenuation of skeletal myopathy. In fact, AET counteracts systemic and local inflammation, neurohumoral exacerbation, and increased oxidative stress, which contribute to skeletal myopathy in HF [20–28]. However, the mechanisms underlying the benefits of AET in counteracting skeletal myopathy are a topic of major interest under current investigation.

Since both pharmacological and nonpharmacological therapies have a direct and/or indirect impact on skeletal myopathy, this review aims to provide an overview of these therapeutic approaches in counteracting skeletal myopathy in HF.

## 2. Impact of Neurohumoral Blockade in HF-Induced Skeletal Myopathy

Sustained sympathetic hyperactivity and RAS activation are commonly associated with the pathogenesis of HF [6]. Despite the changes in cardiac tissue being causal and major in HF, skeletal muscle abnormalities are also affected by neurohumoral overactivation. Even though the beneficial effect of neurohumoral blockade on cardiac muscle is undeniable, there is paucity of data about its effects on skeletal myopathy.

In this section, we will discuss the impact of *β*-blockers and RAS inhibition on skeletal myopathy considering their potential direct and/or indirect effects.

### 2.1. *β*-Adrenergic Receptor Signaling in Skeletal Muscle: Effects of *β*-Blockade in HF

Sympathetic hyperactivity is a hallmark of HF associated with poor prognosis being an independent predictor of mortality [6]. Over the last few years, our group has demonstrated that sympathetic hyperactivity contributes to skeletal myopathy in a mouse model of HF and in patients with HF [15, 20, 29].

In skeletal muscle, the sympathetic activity is mediated by *β*-adrenergic receptors (*β*-AR). These adrenergic receptors are expressed on the membrane of skeletal muscle cells in a proportion of 90 : 10 for subtypes *β*_2_-AR and *β*_1_-AR, respectively [30–32]. Some studies suggest that *β*_3_-AR subtype can also be expressed in skeletal muscle, in a smaller percentage [33, 34].

The *β*-AR density in skeletal muscle can be different depending on the fiber type predominance. Type I fibers express two to three times more *β*-AR than type II fibers [35–37], which corroborates the highest density of *β*_1_-ARs and *β*_2_-ARs in oxidative skeletal muscles when compared to glycolytic ones [32, 37–40]. In fact, Jensen et al. [41] observed that rat* soleus* muscle (predominantly oxidative metabolism and mainly comprised with type I fibers) has a higher density of *β*-ARs than white gastrocnemius (mainly comprised with type II fibers). However, the functional significance of this difference in *β*-AR density between oxidative and glycolytic muscles has not been fully understood, since the *β*-AR responsiveness to *β*-agonists appears to be greater in glycolytic than oxidative muscles [38, 42].

**Figure 1:**
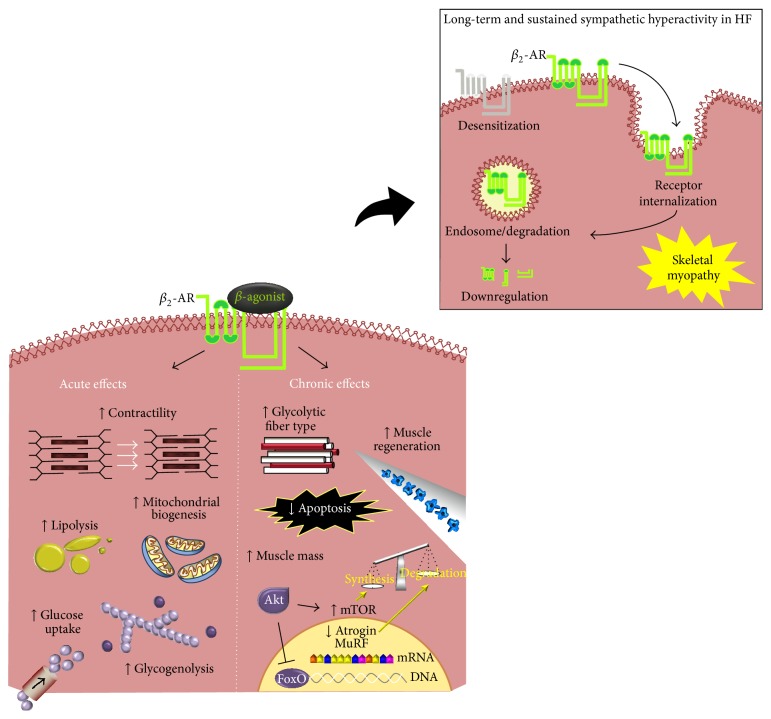
Effects of *β*-adrenergic receptor activation in skeletal muscle. In skeletal muscle, sympathetic activity is mediated mainly by *β*_2_-adrenergic receptors (*β*_2_-ARs) and leads to beneficial acute and chronic effects on muscle metabolism, function, and mass. In this sense, keeping *β*_2_-ARs signaling in early stage HF might be reasonable since it can delay skeletal myopathy. In contrast, long-term and sustained sympathetic hyperactivity (figure inset) in severe heart failure exerts toxic effects on skeletal muscles leading to *β*_2_-AR desensitization/downregulation and loss of function, which will further aggravate skeletal myopathy. In this case, the use of selective (acting on *β*_1_-AR) versus nonselective (acting on both *β*_1_-AR and *β*_2_-AR) *β*-blockers to counteract skeletal myopathy needs further investigation. Akt: protein kinase B, Atrogin-1/MAFbx: muscle atrophy F-box protein, MuRF-1: muscle RING-finger protein-1, FoxO: forkhead family of transcription factors, and mTOR: mammalian target of rapamycin.

Even though the acute and chronic effects of sympathetic activation in skeletal muscle are well known, the role played by sympathetic overactivation in skeletal muscle associated with chronic diseases, such as HF, needs to be better clarified. In this sense, direct acute effects of *β*_2_-AR activation in skeletal muscle include increased lipolysis [43, 44], glycogenolysis [45, 46], glucose uptake [47, 48], and increased contractility [49, 50]. Furthermore, *β*_2_-AR activation has recently emerged as a potential signaling pathway involved in mitochondrial function, biogenesis, and dynamics in skeletal muscle and in other tissues [51–60] (Figure 1). Different from acute *β*_2_-AR activation, the response of skeletal muscle to chronic *β*_2_-AR activation leads to decreased apoptosis [61], improved muscle regeneration [62], increased skeletal muscle strength, a shift toward type II glycolytic fibers, and a pronounced increase in skeletal muscle mass [63–65] (Figure 1). In fact, chronic activation of *β*_2_-ARs leading to skeletal muscle hypertrophy is well described in several studies using *β*-AR agonists, such as clenbuterol, formoterol (selective *β*_2_-AR agonists), and isoproterenol (a nonselective *β*-AR agonist) administration in healthy and atrophic animals [58, 61, 64–67]. Although the signaling pathways responsible for these muscle anabolic effects of chronic *β*_2_-AR activation are poorly understood, they have been associated with an inhibition of proteolysis (calcium-dependent proteolysis and ATP dependent proteolysis) and an activation of protein synthesis signaling pathways (mainly protein kinase B-Akt/mammalian target of rapamycin-mTOR signaling pathway) [58, 65, 68, 69].

Considering the hypertrophic effects of chronic *β*-AR activation, some authors have suggested *β*-AR agonists as a pharmacologic therapy in counteracting cardiac cachexia in late 80's decade onwards. Although these studies have observed some direct beneficial effects of *β*-AR agonists on muscle mass in HF, tachycardia was reported as a main side effect [70]. This might be due to the *β*_1_-AR related cardiac effect, and the use of specific *β*_2_-AR agonists would be more efficient in counteracting, at least, some features of cardiac cachexia [71]. In fact, we have observed that mice lacking *β*_2_-AR display more pronounced exercise intolerance and a more severe skeletal muscle atrophy after HF induced by myocardial infarction when compared with control mice [72]. Therefore, it is possible that, in early stages of the cardiac disease, increased sympathetic activity through the activation of *β*_2_-AR could be able to delay the onset of muscle proteolysis. This seems to be the case in our mice model of sympathetic hyperactivity induced HF. At 3 months of age, these mice display increased sympathetic activity with no signs of HF associated with* plantaris* hypertrophy [29]. This hypertrophic response is mediated by *β*_2_-AR activation since mice lacking *β*_2_-AR display no hypertrophic response to chronic isoproterenol delivery (15 days) [29]. It is of interest that when sympathetic hyperactivity persists and HF aggravates in our mice model,* plantaris* atrophy and skeletal myopathy are observed [72]. Therefore, while acute and chronic activation of *β*_2_-AR by *β*_2_-agonists seems to counteract skeletal myopathy in early stages of the disease, long-term and sustained activation of *β*_2_-AR aggravates skeletal myopathy in HF, which might be related to *β*_2_-AR desensitization and downregulation reducing its anabolic effects.

Studies have shown that *β*_2_-ARs are able to internalize into the cytoplasm of skeletal muscle cell after their activation with *β*_2_-AR agonists. Rothwell et al. [73] demonstrated that prolonged clenbuterol treatment reduces by 65% the density of *β*_2_-AR in skeletal muscle, which suggests receptor internalization and further downregulation. Jensen et al. [41] also showed that 50% of *β*_2_-ARs undergo internalization in skeletal muscle after 30 minutes of isoproterenol treatment. Additionally, 80% of the internalized receptor recycles, returning to the cell membrane. However, it is not clear whether the sensitivity of recycled receptors to the agonist is preserved or not. Therefore, these data highlight the differences in skeletal muscle response to short-term and long-term/sustained *β*_2_-AR stimulation as aforementioned. This is of particular importance if one considers that long-term sustained activation of *β*_2_-AR by sympathetic hyperactivity in HF might lead to *β*_2_-AR downregulation and loss of function, which will further aggravate skeletal myopathy in HF. Indeed, more studies are needed to test whether the usage of nonselective *β*-blockers (blocking *β*_2_-AR in skeletal muscle) in HF therapy would be beneficial or detrimental to counteract skeletal myopathy.

### 2.2. Role of Renin Angiotensin Aldosterone System in Skeletal Muscle: Effects of Its Inhibition in HF

The inhibition of RAS has been demonstrated as an effective pharmacological therapy in HF. The beneficial effects of RAS inhibition in HF include improved clinical status and quality of life and survival paralleled by reduction in neurohumoral activation and hospitalization [74–76].

Angiotensin II (Ang II), the main effector molecule of the RAS (canonical axis), is processed by angiotensin converting enzyme (ACE) from inactive angiotensin I, which is responsible for vasoconstriction, proliferation, and proinflammatory effects [77]. Increased Ang II is a hallmark of HF, and the clinical use of ACE inhibitor or AT_1_ receptor blockade is an obligatory HF therapy, reducing both systemic and local RAS deleterious effects on cardiovascular system [78]. It is of interest that ACE inhibition attenuates body weight loss associated with impaired survival in HF patients [79]. Taking into consideration that body weight loss in HF is also associated with skeletal muscle atrophy [5, 80, 81], the attenuated body weight loss by ACE inhibitors in HF might be due to a reduced loss of muscle mass.

Several studies have demonstrated a detrimental role of Ang II in skeletal muscle, either independently or combined with the systemic RAS activation [82, 83]. A pioneer study by Brink and coworkers showed that Ang II infusion by osmotic minipumps in rats induces a significant body weight loss through a reduction of food intake and decreased circulating insulin-like growth factor-I (IGF-I), which are completely prevented by losartan treatment (AT_1_ receptor blocker) [84]. In fact, Ang II leads to muscle wasting inducing protein breakdown and decreasing IGF-I signaling in skeletal muscle [85–87]. To further confirm the role of Ang II in IGF-I signaling in skeletal muscle, specific overexpression of IGF-I (MLC/mIGF-I mice) is able to inhibit Ang II-induced skeletal muscle wasting through activation of Akt/FoxO (forkhead box O) pathway and inhibition of E3 ubiquitin ligase Atrogin-1/MAFbx mRNA levels [88–91].

Besides its effects on muscle tissue, Ang II inhibits skeletal muscle stem (satellite) cell proliferation, leading to reduced muscle regenerative capacity [92]. In fact, AT_1_ receptor is highly expressed in satellite cells and its activation leads to depleted basal pool of satellite cells, which is blunted by AT_1_ receptor blockade [92]. Even though little is known about the skeletal muscle regenerative capacity in HF-induced muscle wasting, another atrophic state, such as aging sarcopenia (muscle wasting with aging), is associated with a reduced skeletal muscle regenerative capacity associated with a decline in satellite cell function and/or number [93]. Thus, Ang II-mediated inhibition of skeletal muscle regeneration may play a significant role in muscle wasting induced by chronic diseases, such as HF. Therefore, future studies should be addressed to test whether Ang II would induce a potential deficit of skeletal muscle regeneration in HF. Indeed, the effects of RAS inhibition on satellite cells function and number in HF have not been addressed so far.

In addition to Ang II direct effects on skeletal muscle and muscle satellite cells, indirect effects of Ang II regulating circulating hormones, cytokines, and metabolic effectors besides ROS formation also affect muscle wasting. The AMP-activated protein kinase (AMPK) is a key regulator of energy status acting as a metabolic energy sensor modulating glucose and lipid metabolism. Ang II blocks AMPK activity and reduces muscle mass [94]. Conversely, AMPK activation by AICAR reverses Ang II-mediated increased E3 ubiquitin ligases mRNA levels [93]. Other indirect effects of Ang II in skeletal muscle are activation of glucocorticoid-induced muscle breakdown [88] and NADPH oxidase induced ROS formation in skeletal muscle [11, 94–96]. In fact, Ang II-induced muscle wasting was inhibited by NADPH oxidase subunit in p47phox deficient mice [91]. Finally, Ang II increases interleukin-6 (IL-6) cytokine levels leading to an imbalance in protein synthesis : degradation ratio by inhibiting IGF-I/Akt/mTOR signaling while activating ubiquitin-proteasome system (UPS) and caspase-3 promoting muscle wasting [97].

Considering that Ang II plays a role in weight body loss and muscle wasting in HF [79], ACE inhibitors are recommended to partially counteract these effects [98]. Indeed, pharmacological therapy with RAS inhibition in HF patients increases exercise tolerance and quality of life, which might be related to an attenuated skeletal myopathy [99, 100]. In fact, HF treatment with perindopril (ACE inhibitor) increases respiratory muscle strength in humans [101] and partially prevents skeletal muscle dysfunction induced by myocardial infarction in rats [102]. The same has been observed for AT_1_ receptor antagonists attenuating, at least in part, HF-induced reduced skeletal muscle force. Telmisartan increases muscle force generation and endurance performance by activation of peroxisome proliferator-activated receptor gamma- (PPAR*δ*-) AMPK signaling in transgenic mice [103]. Azilsartan medoxomil, a new AT_1_ receptor antagonist, induces Akt phosphorylation and glucose uptake in Sprague-Dawley rats [104]. Irbesartan protects tibial muscle from apoptosis-dependent atrophy in monocrotaline HF model. In myocardial infarcted mice, muscle wasting paralleled by reduced satellite cells numbers is partially inhibited by candesartan treatment [92].

Another important effector of canonical RAS axis activation is the aldosterone, which is also involved in muscle atrophy in HF [105, 106]. Interestingly, spironolactone administration (a mineralocorticoid antagonist) decreased skeletal muscle apoptosis and increased muscle contractility, improving exercise capacity [107]. Therefore, these data provide evidence for a pivotal role of aldosterone in inducing muscle atrophy in HF and mineralocorticoid antagonism as a potential therapy for counteracting skeletal myopathy.

It is important to highlight the complexity of RAS on skeletal muscle mass regulation, since there is a paradoxical “protective arm” in RAS system. While the most deleterious effects of Ang II-induced muscle wasting are mediated via AT_1_ receptors, AT_2_ receptor triggers beneficial effects on muscle regeneration in both* in vivo* and* in vitro* models [108]. Indeed, AT_2_ receptor antagonist reduces regenerating myofiber size and myoblast differentiation marker (e.g., myogenin and embryonic myosin heavy chain, eMHC) mRNA levels [108].

A noncanonical protective axis of RAS involves the peptide Ang-(1–7). Ang-(1–7) is synthesized directly from Ang II or indirectly from Ang I by an ACE homolog enzyme (ACE2), which in turn binds into the Mas receptor [109, 110]. ACE2 is expressed uniformly within* soleus* and* plantaris* muscles [111]. The endogenous RAS axis has opposite effects, when compared to the classical signaling pathway. It triggers vasodilation and improves skeletal muscle metabolism, preventing the fibrosis and apoptosis processes [109, 112].

**Figure 2:**
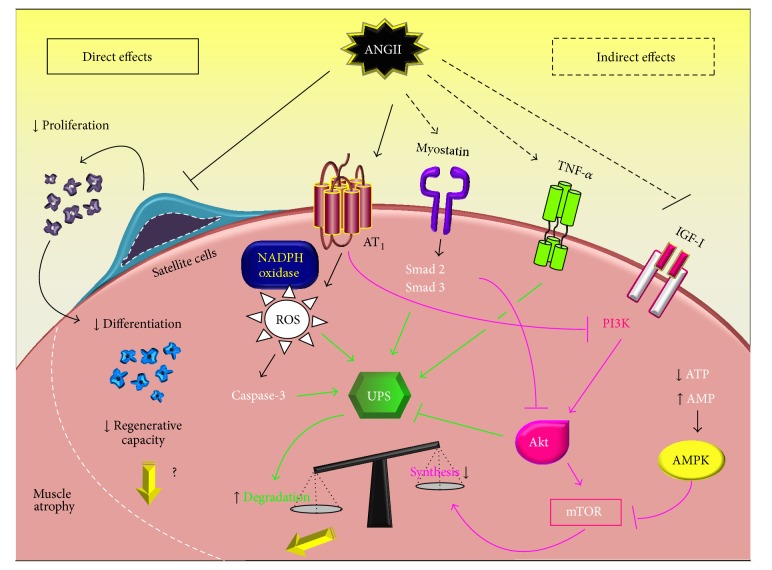
Direct and indirect effects of Ang II on skeletal muscle mass. The direct effects of angiotensin II (Ang II) in skeletal muscle include increases ROS production via AT_1_ receptor and NADPH oxidase activation, which results in activation of UPS and protein degradation. The indirect effects of systemic Ang II are mediated by increased ROS induced caspase-3 besides enhanced TNF-*α* and myostatin levels. Ang II also directly suppresses protein synthesis via AT_1_ receptor inhibiting PI3K and by indirect mechanisms via Ang II attenuating IGF-I while increasing myostatin levels. Ang II-induced muscle wasting can also result from impaired muscle regeneration by Ang II-induced inhibition of skeletal muscle stem (satellite) cell proliferation and function. It is important to highlight that these multiple direct and indirect mechanisms involving Ang II-induced muscle wasting are potential mediators of cardiac cachexia. PI3K: phosphoinositide 3-kinase, Akt: protein kinase B, UPS: ubiquitin-proteasome system, mTOR: mammalian target of rapamycin, TNF-*α*: tumor necrosis factor-alpha, IGF-I: insulin-like growth factor-I, ATP: adenosine triphosphate, AMP: adenosine monophosphate, AMPK: AMP-activated protein kinase, NADPH oxidase: nicotinamide adenine dinucleotide phosphate-oxidase, and ROS: reactive oxygen species.

Taken together, pharmacological therapy with inhibitors of RAS (canonical axis) has demonstrated some positive outcomes in skeletal myopathy of HF patients, such as a partial attenuation in exercise intolerance and muscle wasting. The relative contribution of direct* versus* indirect beneficial effects of RAS inhibition on skeletal myopathy in HF remains unclear. This seems to be (canonical axis) direct and indirect effects on skeletal muscle as can be seen in Figure 2. In parallel, therapies that increase RAS noncanonical axis, such as ACE2 and Ang-(1–7), are also of great clinical relevance for attenuating skeletal myopathy in HF. In this sense, AET has emerged as a potential therapy modulating both canonical and noncanonical axis of RAS (for details, see Section 3.2).

## 3. Impact of Aerobic Exercise Training on Counteracting HF-Induced Skeletal Myopathy

AET has been recognized as an efficient and safe preventive and therapeutic strategy for cardiovascular diseases [113, 114], as it reduces a number of cardiovascular risk factors [115, 116] and improves peak oxygen uptake (peak VO_2_), exercise tolerance, and quality of life [25, 117]. Indeed, both European [118] and American [119] guidelines have agreed upon the recommendation of AET for all stable outpatients, in addition to optimal pharmacological therapy. Several beneficial effects of AET on HF have been demonstrated in heart, endothelium, and skeletal muscle to name a few [120]. It is worth mentioning that the responsiveness of skeletal muscle to AET is far higher than that to pharmacological therapy, which highlights AET as a potent strategy to counteract skeletal myopathy in HF. Indeed, the impact of AET on skeletal muscle is related to several aspects of muscle function as it improves metabolic and contractile properties besides attenuating muscle wasting, as we are going to discuss below.

### 3.1. Muscle Metabolic and Contractile Responses

Skeletal myopathy leads to several muscle metabolic changes in human and animal HF [121–128], such as a switch toward type II glycolytic fibers and decreased mitochondrial density and function (e.g., decreased cytochrome oxidase activity and other oxidative enzymes). Altogether, these changes trigger a reduced aerobic capacity and exercise intolerance [121, 123, 125, 128]. In fact, HF patients display delayed availability of phosphocreatine during exercise, anticipating muscle fatigue [124]. Indeed, a decrease in protein expression of a potent regulator of mitochondrial biogenesis, PGC-1*α* (i.e., peroxisome proliferator-activated receptor gamma), is observed in rat HF [122, 126, 127]. These metabolic changes in skeletal muscle contribute to increased muscle fatigability and lactate accumulation during exercise in HF.

AET has been considered an effective strategy in modulating muscle metabolic changes induced by HF. In fact, AET increases peak VO_2_ and exercise tolerance, which is related to energy production and utilization efficacy. Regarding substrate supply, AET increases muscle phosphocreatine availability and resynthesis [129, 130]. Indeed, muscle ATP levels are increased by AET due to improved oxidative enzyme activities and increased mitochondrial content. These improvements in muscle substrate supply and uptake are optimized by the enhanced blood supply to skeletal muscle, as AET prevents HF-induced capillary rarefaction and endothelial dysfunction [125, 131–133]. Additionally, AET promotes a “reshift” toward more oxidative type I fibers, which are more resistant to muscle fatigue [29, 134].

Another important change induced by HF is a significant impaired muscle contractility associated with changes in Ca^2+^ handling, which will further affect muscle strength and resistance to fatigue. In this sense, HF in rats decreases skeletal muscle sarcoplasmic Ca^2+^ levels associated with reduced rate of sarcoplasmic reticulum Ca^2+^ release [135] and reuptake [123]. These findings are extended to human HF by Middlekauff et al. [136], who verified reduced Ca^2+^ release and reuptake associated with decreased dihydropyridine receptors and sarco(endo)plasmic reticulum Ca^2+^-ATPase (SERCA) 2a protein expression in* vastus lateralis* of HF patients.

AET improves skeletal muscle Ca^2+^ handling. We have previously demonstrated that AET improves the net balance of Ca^2+^ handling proteins in HF mice involved in sarcoplasmic Ca^2+^ release and reuptake in* soleus* and* plantaris* muscles leading to improved skeletal muscle function [137]. Interestingly, endurance leg extension training also reduces Ca^2+^ leaking by ryanodine receptors in* vastus lateralis* of HF patients [138].

Taken together, data from the literature provide evidence for AET as a strategy of paramount importance to prevent muscle metabolic and contractile dysfunction in HF.

### 3.2. Neurohumoral and Muscle Mass Regulation

Skeletal muscle loss is considered an independent predictor of morbidity and mortality in HF patients [139]. In fact, muscle wasting is triggered by neurohumoral overactivation and increased proinflammatory cytokines [27, 140, 141] being associated with impaired strength and peak VO_2_ [139, 142]. So far, no specific treatment is available for muscle wasting in HF. Therefore, adjuvant nonpharmacological therapies as nutritional supplementation and AET have been encouraged. Indeed, AET is efficient in counteracting skeletal myopathy in HF by improving skeletal muscle function (direct effect) or by attenuating cardiac dysfunction and neurohumoral hyperactivity (indirect effect).

Regarding neurohumoral overactivation, AET reduces muscle sympathetic nerve activity, which is associated with an improved clinical outcome [24, 25]. In fact, Roveda et al. [25] have demonstrated that a 4-month period of moderate AET leads to a significant reduction in muscle sympathetic nerve activity in HF patients, returning to the values of age-matched healthy controls. The mechanisms underlying the reduction in sympathetic hyperactivation by AET are still a topic under current investigation. The potential candidates underlying the reduced sympathetic nerve activity by AET include afferent autonomic control coordinated by arterial baroreceptors, cardiopulmonary receptors, ergoreceptors, and chemoreceptors [143–145]. AET can improve muscle metaboreflex and mechanoreflex control of muscle sympathetic nerve activity in HF animal models and human HF [146–148]. In fact, we have recently observed that AET improves metaboreflex and mechanoreflex associated with changes in cyclooxygenase pathway [149]. Indeed, AET plays an important role in the control of cardiovascular reflexes by the central nervous system. In this sense, AET seems to modulate projections arising from hypothalamic paraventricular nucleus (mainly, peptidergic hypothalamic preautonomic neurons) that converge to nucleus tractus solitarii [147]. Additionally, reduced AT_1_ receptors of angiotensin II in nucleus tractus solitarii and normalized ACE and ACE2 levels in the brain of HF animal models have been proposed as major mechanisms of reducing sympathetic activity by AET [148, 150].

AET reduces circulating catecholamine levels in both HF patients and animal models [26, 27]. AET improves capillary density and muscle redox balance in a sympathetic hyperactivity induced HF mice model [20, 29]. This is of particular interest, since sympathetic hyperactivity directly affects skeletal muscle [29, 137] promoting redox imbalance [7, 151], chronic vasoconstriction [14, 15], and increased muscle norepinephrine and proinflammatory cytokine levels [140, 141].

RAS hyperactivity is also involved in skeletal myopathy in HF primarily by activation of Ang II, increasing ROS generation, protein degradation, and apoptosis as aforementioned in Section 2.2. Interestingly, we have previously demonstrated that AET reduces serum Ang II levels besides increasing muscle Ang-(1–7), Ang-(1–7)/Ang II ratio, and Mas receptor gene expression [28]. Additionally, reduced serum Ang II levels were related to decreased sympathetic activity in HF, favoring the redox balance [7].

Neurohumoral overactivation is also associated with increased circulating/muscle proinflammatory cytokine concentrations and muscle redox imbalance, which are directly involved in muscle catabolism. In fact, increased circulating TNF-*α* levels (a proinflammatory cytokine) are observed in patients with reduced skeletal muscle cross-sectional area and muscle weakness [152]. Moreover, the increased muscle TNF-*α* expression contributes to the local protein degradation, worsening muscle function and metabolism. The effects of TNF-*α* on HF-related skeletal myopathy are mediated through the activation of a family of transcription factors known as nuclear factor kappa B (NF-*κ*B), which regulate UPS [153]. NF-*κ*B activation leads to increased expression of the E3 ubiquitin ligases MuRF-1 and Atrogin-1 [153] and muscle proteolysis, leading to atrophy. Interestingly, AET reduces serum TNF-*α* levels [22] and plasma inflammatory markers (e.g., soluble cell adhesion molecule-1, soluble vascular cell molecule-1, and macrophage chemoattractant) [154] in trained HF patients. This response is paralleled by reduced muscle atrophy and improved muscle strength. Indeed, AET also reduces muscle expression of proinflammatory cytokines in human HF [23] while preventing TNF-*α* mediated diaphragmatic weakness in mice [155].

Increased TNF-*α* levels in HF triggers an increase in ROS production [156], which will ultimately lead to protein degradation by the UPS [157, 158]. We have observed that AET reduces moderate (lipid hydroperoxidation) and severe (carbonylated proteins) muscle oxidative stress markers and restores antioxidant activity (e.g., superoxide dismutase) in exercise trained HF animals and patients [20, 29, 159]. Interestingly, we have observed HF-induced NADPH oxidase hyperactivity, an important source of superoxide in skeletal muscle [11]. Such hyperactivity displayed a positive correlation with muscle atrophy and increased UPS activity.

UPS is considered the main proteolytic system responsible for disposal of damaged proteins in skeletal muscle [160], which is upregulated in HF [20, 161]. Indeed, several studies have demonstrated that skeletal muscle atrophy is mediated by the UPS overactivation [162, 163]. Although the UPS involves the concerted actions of many proteins, the key enzyme in this system is E3 (ubiquitin ligase), which couples activated ubiquitin with lysine residues on protein substrates and confers specificity to the system [158, 164]. Two muscle-specific E3 ubiquitin ligases, Atrogin-1 and MuRF-1, are increased transcriptionally in skeletal muscle under various atrophying conditions including HF [161, 164–166], making them excellent markers of muscle atrophy [167]. In contrast, AET reduces Atrogin-1 mRNA levels and proteasome activity, in animal and human HF, being an efficient strategy to prevent UPS overactivation induced by HF [21, 159, 168]. Additionally, Souza et al. [126] observed that AET by increasing muscle PGC-1*α* expression levels prevented Atrogin-1 and MuRF-1 upregulation in an aortic stenosis model of HF rats, reinforcing the anticatabolic effect associated with increased muscle PGC-1*α* levels [169].

Besides protein degradation, protein synthesis also plays an essential role in maintaining muscle mass [170]. Several signaling pathways are involved in protein synthesis, such as IGF-I/Akt/mTOR. In fact, IGF-I muscle levels are reduced in human HF [171]. Therefore, activation of the IGF-I/Akt/mTOR signaling pathway can be considered a good strategy to counteract HF-induced muscle wasting and cardiac cachexia. Muscle-specific IGF-I transgene expression or gene transfer in hindlimb muscles sustains muscle hypertrophy [172] and prevents muscle wasting in rodent models of muscle atrophy including Duchenne muscular dystrophy [173], dexamethasone injection [174], cast immobilization [175], Ang II infusion [88], and chronic HF [176]. Furthermore, another strategy to increase the expression of the gene encoding IGF-I is through AET [177, 178], which attenuates the reduced muscle IGF-I expression in HF patients [176, 179].

Altogether, these results suggest that AET reestablishes skeletal muscle homeostasis attenuating muscle wasting. This is crucial since muscle wasting in HF is related to a poor prognosis and reduced quality of life [139].

**Figure 3:**
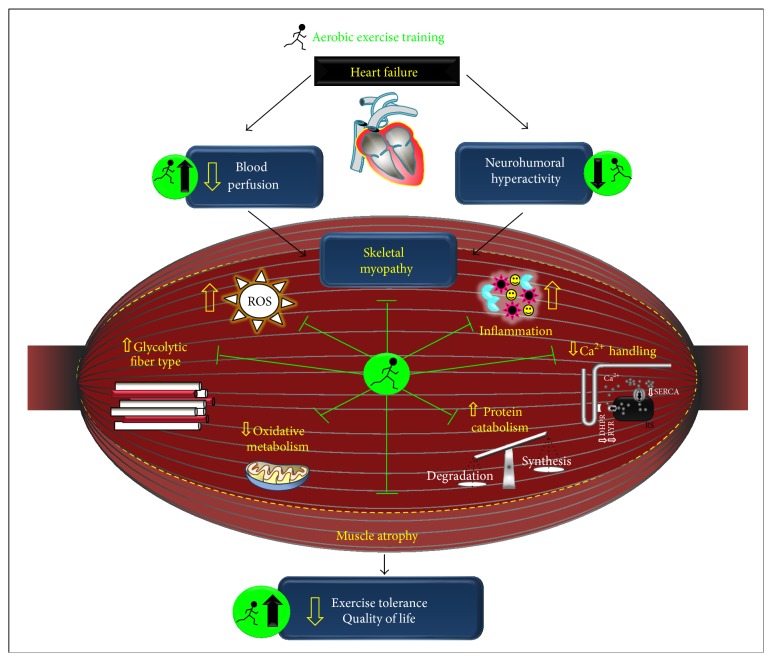
Effects of aerobic exercise training in counteracting heart failure-induced skeletal myopathy. Neurohumoral hyperactivity and reduced blood perfusion associated with heart failure contribute to skeletal myopathy, which is characterized by muscle prooxidant and inflammatory state associated with muscle contractile dysfunction and atrophy, exercise intolerance, and reduced quality of life. These responses are associated with impaired Ca^2+^ handling, reduced protein synthesis paralleled by increased proteolysis. Note that aerobic exercise training counteracts most of the features involved in skeletal myopathy (illustrated by filled arrows and ⊥). ROS: reactive oxygen species, RYR: ryanodine receptor, DHPR: dihydropyridine receptor, and SERCA: sarcoplasmic reticulum Ca^2+^ ATPase.

This seems to be AET effects on skeletal muscle in HF as can be seen in Figure 3.

## 4. Similarities and Differences between Aerobic Exercise Training and Neurohumoral Blockade in HF-Induced Skeletal Myopathy

Over the last few decades, the therapeutic approach most commonly used in HF has been the neurohumoral blockade, which is currently mandatory [180]. *β*-blockers and RAS inhibitors improve cardiac function, trigger reverse remodeling, and are directly related to reduced morbidity and mortality [181]. Furthermore, these drugs cause systemic effects, such as reduced inflammatory response and oxidative stress contributing to both cardiac and skeletal muscle improvements [76, 93, 181, 182]. These systemic effects could minimize the impairments caused by HF in skeletal muscle, improving muscle mass regulation, metabolism, and function. However, the effects of a neurohumoral blockade on skeletal muscle are still controversial, as already discussed in Section 2.1. The blockade of *β*-AR modifies the skeletal muscle metabolism and impairs exercise tolerance, resulting in increased perceived exertion, lower VO_2_max, and work rate in hypertensive as well as in healthy individuals [183–185]. These responses have been mainly associated with pharmacological properties of different generations of *β*-blockers. Nonselective *β*-blockers, which also antagonize *β*_2_-AR, may worsen these effects even more [186, 187]. Nevertheless, nebivolol, which possesses vasodilative properties mediated by NO production, does not impair exercise capacity in healthy individuals [186, 188]. Indeed, Dalla Libera et al. [189] verified a decreased apoptosis and proinflammatory cytokines, preventing fiber shift and protein oxidation in skeletal muscle of HF rats treated with nebivolol. These positive changes could produce a favorable impact on exercise capacity and skeletal myopathy in humans [189].

RAS has also been inhibited to minimize the neurohumoral overactivation and improve cardiac function in HF. Moreover, ACE inhibitors have been associated with beneficial effects on skeletal muscle, such as improved muscle glucose uptake and mitochondrial function and a modulation of IGF-I, which is related to skeletal muscle trophicity [102, 190]. Additionally, in hypertensive patients treated with ACE inhibitors, an increased skeletal muscle cross-sectional area and a slower decline in walking speed are observed when compared to patients under other antihypertensives [99]. Another study [100] observed a smaller reduction in muscle strength and improved quality of life in elderly patients submitted to an ACE inhibitor treatment. Despite these results, it is not possible to affirm that these effects would revert skeletal myopathy in HF. Indeed, whether ACE inhibitor responses are a result of a direct effect on skeletal muscle or secondary to systemic changes (improved cardiac function and reduced proinflammatory cytokines and oxidative stress) remains to be determined.

**Figure 4:**
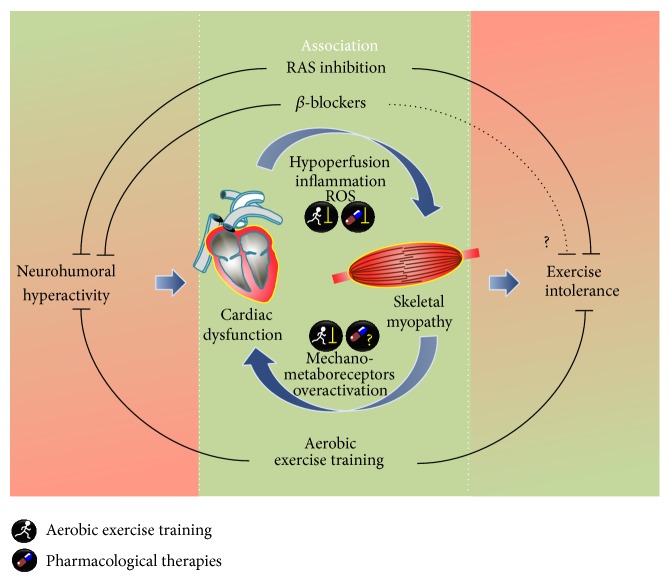
Similarities and differences between aerobic exercise training and neurohumoral blockade in heart failure-induced skeletal myopathy. Skeletal myopathy in heart failure plays a major role in exercise intolerance. Neurohumoral hyperactivity is associated with the pathogenesis of heart failure and also affects skeletal muscle by increasing inflammatory response and oxidative stress and decreasing muscle perfusion. In this perspective, neurohumoral blockade has an important indirect effect on attenuating skeletal myopathy by improving cardiac function and reducing neurohumoral hyperactivity (thick solid lines and ⊥). The efficacy of direct effects of *β*-blockade and renin angiotensin aldosterone system inhibition on skeletal muscle still need to be clarified, as exercise intolerance remains in heart failure patients (thin solid lines and ⊥), mainly the ones under *β*-blocker therapy (dashed lines and ⊥). In contrast, aerobic exercise training has been considered the most effective strategy to counteract skeletal myopathy and to improve exercise tolerance in heart failure. Therefore, combined neurohumoral inhibition and aerobic exercise training are of great clinical interest in heart failure therapy.

AET has been considered the most effective strategy to counteract skeletal myopathy in HF. As aforementioned, AET improves several aspects involved in skeletal muscle function regulation, such as substrate supply availability, oxidative enzyme activities, and mitochondrial content (metabolism) [125, 129]; Ca^2+^ release and reuptake (contractility) [137, 138]; and inflammation response and redox balance, reducing skeletal muscle degradation (muscle mass regulation) [20, 21]. Altogether, these AET effects contribute to increasing muscle strength and to improving exercise tolerance and quality of life in HF. Although AET also causes improvements in cardiac function by reducing systemic inflammation and neurohumoral overactivation, there are no studies that provide evidence for a direct link between the reduction of mortality and AET beneficial effects. In contrast, neurohumoral pharmacological blockade reducing HF mortality changed the HF therapy in late 80's [180]. In fact, AET is considered an adjuvant therapy in HF, contributing to minimizing systemic effects and to improving or reverting skeletal myopathy. In the same way, more studies should be conducted to elucidate the role of neurohumoral blockade, *β*-blockers, and RAS inhibitors, in counteracting skeletal myopathy, since the effects of neurohumoral blockade on different features of skeletal myopathy are of low magnitude. Therefore, the combined effects of neurohumoral blockade and AET on HF-induced skeletal myopathy are of great clinical interest. In fact, HF mice submitted to combined carvedilol (*β*-blocker) and AET therapies show an integration of distinctly different beneficial effects of isolated therapies on exercise capacity, ventricular function, and remodeling associated with improved Ca^2+^ homeostasis and reduced ventricular oxidative stress [191]. The summary of the association of neurohumoral blockade and AET on skeletal myopathy is depicted in Figure 4.

In conclusion, an association between pharmacological treatment and AET is currently the most efficient strategy to treat the cardiac dysfunction and skeletal myopathy, improving exercise tolerance and quality of life in HF. It is expected for the future that an association between more selective/specific drugs and AET optimizes the treatment, increasing the responsiveness and outcomes.
